# On Short-Time Estimation of Vocal Tract Length from Formant Frequencies

**DOI:** 10.1371/journal.pone.0132193

**Published:** 2015-07-15

**Authors:** Adam C. Lammert, Shrikanth S. Narayanan

**Affiliations:** 1 Computer Science Department, Swarthmore College, Swarthmore, PA, United States of America; 2 Signal Analysis and Interpretation Laboratory, University of Southern California, Los Angeles, CA, United States of America; Northwestern University, UNITED STATES

## Abstract

Vocal tract length is highly variable across speakers and determines many aspects of the acoustic speech signal, making it an essential parameter to consider for explaining behavioral variability. A method for accurate estimation of vocal tract length from formant frequencies would afford normalization of interspeaker variability and facilitate acoustic comparisons across speakers. A framework for considering estimation methods is developed from the basic principles of vocal tract acoustics, and an estimation method is proposed that follows naturally from this framework. The proposed method is evaluated using acoustic characteristics of simulated vocal tracts ranging from 14 to 19 cm in length, as well as real-time magnetic resonance imaging data with synchronous audio from five speakers whose vocal tracts range from 14.5 to 18.0 cm in length. Evaluations show improvements in accuracy over previously proposed methods, with 0.631 and 1.277 cm root mean square error on simulated and human speech data, respectively. Empirical results show that the effectiveness of the proposed method is based on emphasizing higher formant frequencies, which seem less affected by speech articulation. Theoretical predictions of formant sensitivity reinforce this empirical finding. Moreover, theoretical insights are explained regarding the reason for differences in formant sensitivity.

## Introduction

The length of the vocal tract, measured along its midline from the glottis to the lips, is an essential parameter to consider for explaining behavioral variability in speech production. This structural characteristic of the speech production apparatus determines many aspects of the acoustic speech signal and, at the same time, is highly variable across speakers. The vocal tract lengthens throughout development, from an average length of approximately 8 cm at birth, up to 16 cm in adulthood [1–3]. Even between adults, vocal tracts vary from approximately 13 cm to 20 cm in length. These differences are particularly significant when one considers the relatively limited ability of most individuals to modulate vocal tract length, mainly through lip protrusion and laryngeal height. The role of vocal tract length in vowel production variability has been extensively studied and modeled, particularly with regard to the position and spacing of formant frequencies. It has been well-established that longer vocal tracts are associated with lower formant frequencies. This effect is supported theoretically [4, 5], and has been repeatedly confirmed empirically [6, 7]. Recent work to quantify vocal tract length as a source of acoustic variability suggests that it is the second largest source of formant frequency variability overall after phonemic identity, accounting for up to 18% [8].

Given that information about vocal tract length is available in the acoustic signal, it is reasonable to expect that accurate predictions of vocal tract length should be possible from acoustic information alone. Developing a method to produce vocal tract length estimates is easily motivated by the many practical applications that could be found for such a method. For instance, accurate vocal tract length estimation would afford the ability to normalize the acoustic characteristics of vowels for meaningful comparison across speakers. Indeed, vocal tract length normalization (VTLN) is already commonly used in automatic speech recognition (ASR) applications and has been shown to provide significant gains in system performance [9–11]. VTLN techniques typically seek a frequency scale transformation that allows for optimal comparison of spectral features extracted from different speakers. Appropriate transformations are commonly found by optimizing some maximum likelihood criterion over a set of acoustic data [12, 13], although formant alignment has also been investigated [14, 15]. However, because the end goal of such techniques is the improvement of ASR performance, their ability to accurately estimate vocal tract length as a physical quantity has not been rigorously validated on data sets with careful vocal tract length measurements.

Accurate estimation of vocal tract length from the acoustic signal has been addressed in a handful of studies. In developing estimation techniques, the focus has been on the utility of formants as features. Formants are the resonant frequencies of the vocal tract *filter* [4], which is often treated as distinct from the vocal *source*, at the glottis. Almost all such studies have incorporated formants as features. Using formants is attractive because they are readily available in the speech signal—especially lower formants—and because the physical relationship between length and formant frequencies is fairly well-understood. One difficulty with relying on formants is that vocal tracts of different lengths can be made to produce the same formant frequencies through deformation of the area function, and formants alone are not enough to recover the area function and normalize out its effects [16]. Some studies have also incorporated the zeros as additional features, which are sufficient to recover an approximation to the area function when combined with formants [17], although they are not directly available in the acoustic signal. Other studies have utilized resonance bandwidths in addition to formant frequencies. Bandwidths are available in the acoustic signal, but they can be difficult to measure robustly. The most substantial differences, though, between the various proposed techniques have been in the form of the models used for estimation, and whether those models lend themselves to closed-form or iterative solutions.

Paige and Zue [18] developed a closed-form estimator based on an approximate relationship between resonant frequencies and the parameters of a band-limited approximation to the area function. Length was determined by minimizing a criterion that identifies the most uniform tube that might have produced a given formant structure. This estimator was initially tested using the first three poles and zeros as input, and was found to produce absolute errors in the range of 3.5 to 11.5% of total vocal tract length on a small data set. Using an iterative method that effectively increases the number of poles and zeros by estimating their frequencies, errors dropped down to the range of 0.6 to 4.9%. Subsequent studies have found that, when either of these estimators is applied to only a small number of formants, the performance drops dramatically [19, 20].

Wakita [21] used the same uniformity criterion to develop an iterative algorithm that takes a set of formant frequencies and bandwidths as input. This method was able to produce absolute errors in the range of 1.6 to 8.6% of total vocal tract length on a small data set of full vowels. It should be noted that the literature repeatedly remarks that there is no compelling theoretical reason behind the uniformity criterion, despite the fact that it provides good performance in practice. Indeed, additional experiments by Necioglu [20] have provided additional empirical evidence that this criterion, and several variants of it, result in reasonable estimation performance.

Wakita [21] also suggested that several closed-form estimation techniques are possible to build, using only formant frequency information as input features. These suggestions were based on the observation that higher formants tend to be less affected by speech articulation (e.g., changes in the area) and therefore reflect vocal tract length more reliably. Specifically mentioned were using the fourth formant alone, and taking the mean length estimate from the fourth and higher formants. Adding successively higher formants provided increasing performance. In order for these closed-form estimators to be competitive with the iterative algorithm, however, it was necessary to utilize the fourth through eighth formants, which would be very difficult to estimate in general.

Kirlin [19] presented a probabilistic formulation of estimating vocal tract length from formant frequencies. By treating formant frequencies as erroneous measurements of a uniform vocal tract’s resonances, Kirlin was able to effectively find closed-form expressions for both the maximum likelihood estimate and the maximum a posterior estimate of vocal tract length given some formant frequencies. When combined with data from the study by Wakita [21], this formulation resulted in perhaps the first estimator designed in a data-driven fashion. Performance of this estimator was shown to be highly competitive with the previously proposed iterative methods when tested on a small data set, which clearly demonstrates the power of a statistical, data-driven approach in designing a closed-form estimator.

Fitch [22] also proposed an estimator based on the spacing of successive formant pairs. As is consistent with theory, if vocal tract length is increased while the area function is held constant, all formant frequencies should lower and the spacing between formants should decrease. This estimator has been shown to correlate well with both vocal tract length and body size in human males [23], and has been used extensively in the literature as a predictor of body size in a variety of other animals, including domestic dog [24], rhesus macaques [22], red deer [25], and colobus monkeys [26].

The present study further examines the possibility of accurate vocal tract length estimation from formant frequencies. Exclusive consideration is given to estimators that use only formant frequencies as input because of their ready availability in the acoustic signal. In keeping with previous work on this topic, it will be assumed that length estimation must be performed on a single short-time analysis window, and that information about speech articulation and phonemic identity is completely unknown. Furthermore, the focus here is on the design and evaluation of closed-form estimators because of the practical (e.g., computational) advantages associated with having a close-form solution, and because the various closed-form estimators mentioned above have not been rigorously evaluated and compared on larger data sets.

In examining vocal tract length estimation with these constraints, the specific goals of this work are as follows: (1) to develop a general framework from the basic principles of vocal tract acoustics for describing estimation methods, (2) to propose a new estimation method that follows naturally from the developed framework, (3) to evaluate this new method using simulated vocal tract data and real human speech data from real-time magnetic resonance imaging (rtMRI), and (4) to provide a theoretical justification for the proposed estimation method based on an examination of the relative sensitivity of different formants to changes in the vocal tract area function.

It is important to note from the outset that there are two perspectives regarding the concept of vocal tract length. The perspective taken here is that vocal tract length is a static characteristic that is inherent to a given speaker. Such a characteristic might be measured, for instance, from a neutral or overall average vocal tract configuration. As such, the present work focuses on the development and evaluation of methods for accurate estimation of a single length parameter per speaker. Vocal tract length defined in this way could be used directly for speech normalization in ASR applications, or could function more generally as a physiologically and perceptually meaningful normalization factor for speech. It could also be used as an invariant speaker characteristic for applications in speaker modeling and identification, or as a correlate of other physiological characteristics (e.g., body size, as in the work by Fitch [22]). The alternative perspective regards vocal tract length as a dynamic characteristic that changes over short timescales (e.g., from phonetic segment to phonetic segment) due to lip rounding, larynx height and even tongue shape. Indeed, this was the perspective taken by Paige and Zue [18] and Wakita [21]. Although the dynamic perspective is not taken in the present work, and accuracies in that regard are not evaluated here, it should be noted that all the methods discussed here will operate in the context of either perspective, and the relationship between both perspectives is discussed at various points throughout the present paper.

The Method section of this paper describes our methodology, including the framework of vocal tract length estimators and the proposed method, as well as the details of acoustic modeling, rtMRI acquisition and experimental setup. In Results & Discussion, the results of the various experiments are described, as well as a discussion of the experimental results and theoretical insights. Final remarks can be found in Conclusion.

## Method

### Vocal Tract Length Estimation Framework

The current approach to length estimation proceeds, as many previous efforts have, from the well-known resonant properties of a tube which is assumed to be lossless and uniform in cross-sectional area along its length, with an idealized radiation impedance and an idealized (i.e., fully and statically closed) glottal termination. These simplifying assumptions are widely made in first-order examinations of vocal tract acoustic characteristics, particularly the assumption of losslessness. As such, they will allow for the development of a general framework for vocal tract length estimation into which previous estimators and the presently proposed estimator will be placed. Assumptions related to the radiation impedance and cross-sectional area are justified by the motivation to create a purely acoustic method of estimation, where no knowledge of the area function can be assumed. Under such constraints, perhaps the most neutral assumption about the area function is that it is uniform. The radiation impedance is assumed to be zero for similar reasons, namely that it depends on the effective radius of the area function at the lips, which is here assumed to be unknown.

Under these assumptions, the length of the vocal tract has a simple relationship with vowel formant frequencies of the form:

$$\begin{array}{c} L=\frac{c}{4\Phi } \end{array}$$ (1)

where *L* is the length of the vocal tract, *c* is the speed of sound. The parameter Φ is defined as the lowest—or, first—resonance frequency of a lossless uniform vocal tract of length *L*. The term *length* will be used throughout the manuscript to refer to distance along the longitudinal axis of the vocal tract, extending from the glottis to the lips. This term contrasts with the term *width*, which will be used to refer to distance perpendicular to the longitudinal axis in the midsagittal plane. The terms *constriction* and *narrowing* will be used to refer to reductions in area in the plane perpendicular to the longitudinal axis of the vocal tract.

For a lossless uniform vocal tract, the parameter Φ is related to formant frequencies by:

$$\begin{array}{c} \Phi =\frac{F_{n}}{\left(2n-1\right)},\;n=1,2,3,\ldots \end{array}$$ (2)

where *n* represents the integer label of the formant frequency. Thus one can easily calculate length of a lossless, uniform vocal tract from any formant frequency using Eqs (1) and (2). In the case of speech, however, the strict assumptions behind Eqs (1) and (2) are not generally applicable, and the relationship expressed in Eq (2) is only approximate. In particular,

$$\begin{array}{c} \hat{L}=\frac{c}{4\hat{\Phi }}, \end{array}$$ (3)

where $\hat{L}$ and $\hat{\Phi }$ are approximations to *L* and Φ.

Any calculation of vocal tract length using Eq (3) with formant frequencies from human speech data is an estimate, and each formant frequency can be considered as a feature that has the potential to provide some information about vocal tract length. It becomes of interest to determine the usefulness of these features and the accuracy of estimates that can be obtained using this model. To that end, it is possible to generalize the linear relationship between Φ and *F*_*n*_ expressed in Eq (2), allowing one to incorporate all formant frequencies as features into a linear combination. In particular,

$$\begin{array}{c} \hat{\Phi }=\frac{\beta_{1}F_{1}}{1}+\frac{\beta_{2}F_{2}}{3}+\frac{\beta_{3}F_{3}}{5}+\cdots +\frac{\beta_{m}F_{m}}{2m-1} \end{array}$$ (4)

up to the highest possible integer formant number, *m*, that can reliably be estimated from the frequency spectrum. Note that, if the assumptions behind Eqs (1) and (2) are correct, then Eq (4) provides a precise value for Φ if any single coefficient *β*_*n*_ = 1 and all others are zero. The same is also true if all coefficients *β*_1…*m*_ = 1/*m*.

This linear model will serve as the basic, generalized framework for estimating Φ from formant frequencies. It is generalized because it allows for information about Φ contained in any formant frequency to be incorporated into the estimate. It is basic because there are several obvious ways to extend the model, including by the addition of terms that raise formant frequencies to higher powers (e.g., $\beta_{n_{2}}F_{n}^{2}$), which would make this a nonlinear model. Perhaps the most obvious extension of this basic model would be to add a constant offset term, *β*_0_, in order to make this a full multiple linear regression model (i.e, one that need not pass through the origin). Extending the model by adding an offset term has a specific motivation stemming from Kirlin’s [19] probabilistic formulation of vocal tract length estimation. This specific extension will be discussed in greater detail in the section entitled results & discussion.

For present purposes the discussion will be confined to the basic, general linear model in Eq (4), which is sufficient to describe a variety of estimation schemes, including many previously proposed estimators of length. In fact, one can incorporate several previously proposed estimators into this framework. It was suggested by Wakita [21] that higher formant frequencies are less sensitive to speech articulation, and would therefore provide more robust estimates of vocal tract length. Using a single formant frequency, *F*_*n*_, to estimate Φ from Eq (4) can be represented by setting coefficient *β*_*n*_ = 1 and all others to zero. Wakita [21] also suggested that averaging together the *p* highest formants might provide an even more robust estimate, which can be represented by setting all coefficients *β*_1_…*β*_*m*−*p*_ to zero and those *β*_*m*−*p*+1_…*β*_*m*_ to 1/*p*.

Fitch [22] proposed an estimator called Frequency Dispersion, based on the average spacing between successive formant pairs:

$$\begin{array}{c} {\hat{\Phi }}_{FD}=\frac{F_{2}-F_{1}}{2(m-1)}+\frac{F_{3}-F_{2}}{2(m-1)}+\cdots +\frac{F_{m}-F_{m-1}}{2(m-1)}. \end{array}$$ (5)

Note that Eq (5) can be considerably simplified, because many of the terms ultimately cancel out. The equation can be eventually re-written as:

$$\begin{array}{c} {\hat{\Phi }}_{FD}=-\frac{F_{1}}{2(m-1)}+\frac{F_{m}}{2(m-1)}. \end{array}$$ (6)

Eq (6) can be made consistent with the general form specified above by setting all coefficients in Eq (4) to zero, except for $\beta_{1}=-\frac{1}{2m-2}$ and $\beta_{m}=\frac{2m-1}{2m-2}$.

Kirlin [19] provided a probabilistic formulation that allowed for explicit maximization of the conditional probability of Φ given some measured formant frequencies: *p*(Φ∣*F*_*n*_). The maximum likelihood estimate of Φ given Kirlin’s formulation is:

$$\begin{array}{c} {\hat{\Phi }}_{MLE}=\frac{\sum_{n=1}^{m}\left(2n-1\right)F_{n}/\sigma_{n}^{2}}{\sum_{n=1}^{m}\left(2n-1\right)/\sigma_{n}^{2}}, \end{array}$$ (7)

where $\sigma_{n}^{2}=\frac{1}{m}\sum_{n=1}^{m}{\left(F_{n}-\Phi (2n-1)\right)}^{2}$. To place this estimator in the current framework, one can easily manipulate the terms to find that

$$\begin{array}{c} \beta_{n}=\frac{{\left(2n-1\right)}^{2}}{\sigma_{n}^{2}\sum_{i=1}^{m}{\left(2i-1\right)}^{2}/\sigma_{i}^{2}}. \end{array}$$ (8)

Working within the framework presented above raises an obvious question: what is the optimal set of coefficients for predicting $\hat{\Phi }$—for instance, what values minimize the least-squared error criterion? One answer to this question can be obtained by setting the coefficients in a data-driven fashion by treating this question as a regression problem. In order to do that, one must have a data set containing a matrix, **M**, where each row contains a set of normalized formant frequencies, that is *F*_*n*_/(2*n* − 1) for *n* = 1…*m*. It is also necessary to have a vector, **Φ**, with the same number of rows as **M**, containing corresponding values of Φ. The desired vector of coefficients, **β**, are then found according to:

$$\begin{array}{c} \beta ={\left(M^{T}M\right)}^{-1}M^{T}\Phi \end{array}$$ (9)

which is a solution to ordinary least-squares regression. The data utilized for this purpose should ideally be gathered from a variety of vocal tract configurations that are representative of speech articulation. Two methods of gathering such data are explored here: acoustic modeling and acquisition of human speech data.

### Acoustic Modeling

Modeling vocal tract acoustics is done in two ways, as described in the following sections: Multi-Tube Model and Perturbation Theory. The first model is based on treating the vocal tract as a series of concatenated tubes [27]. This model, which will be henceforth referred to as the ‘multi-tube’ model, has been well-studied with respect to vocal tract modeling and synthesis [cf. [4, 5, 28, 29]]. The second method is based on perturbation theory, which has a similarly long history in speech production research [5, 30]. These models are utilized for two purposes in the present study. The multi-tube model is used to generate synthetic formant frequencies for experiments in vocal tract length estimation, while both models are used to provide theoretically-motivated explanations for the form of the estimation models. The specifications of these models are explained in the following subsections.

It should be noted that the primary reason for utilizing these models is not to provide highly faithful speech synthesis. Rather, these models are meant to provide correspondence to essential aspects of human vocal tract acoustics, but with certain simplifying assumptions. As stated in section Vocal Tract Length Estimation Framework, these assumptions include idealized geometry, lack of loss and zero radiation impedance. It is important to make these assumptions for a number of reasons. First, they are intended to be consistent with previous work on vocal tract length estimation (e.g., by Wakita [21] and Fitch [22]), which can then be unified into the proposed estimation framework. Because this framework subsequently forms the basis for the proposed estimator, the performance of all the estimators considered can then be meaningfully compared. These assumptions also facilitate the theoretically-motivated insight into the performance of the proposed estimator. Because the assumptions are somewhat strong, the present study includes a set of parallel estimation experiments on real human speech data. Indeed, comparing estimation accuracies on real and synthetic data provides an opportunity—discussed later in the section Data for Estimation Experiments—to assess estimation accuracy as a function of potential mismatch between modeling assumptions and characteristics of the data. It is for all these reasons that the assumptions in the acoustic models are consistent with the assumptions behind the proposed estimator.

#### Multi-Tube Model

The multi-tube model treats the vocal tract as a series of lossless, cylindrical tubes that are concatenated end-to-end. For a given area function, *A*, the formant frequencies can easily be computed by first calculating the reflection coefficients between each pair of adjacent tubes:

$$\begin{array}{c} \Gamma \left(x\right)=\frac{A(x+1)-A(x)}{A(x+1)+A(x)}, \end{array}$$ (10)

where *A*(*x*) is the cross-sectional area of the vocal tract at distance *x* from the glottis. Reflection coefficients are then used to compute the coefficients of the prediction filter polynomial. This is done using Levinson recursion, as described in [31] and implemented in the Matlab^®^ Signal Processing Toolbox^™^ (The MathWorks Inc., version 7.8.0). Finally, the formant frequencies can be found by taking the roots of the prediction filter polynomial (in radians), and converting to Hertz. Formant frequencies are then sorted and assigned integer labels.

#### Perturbation Theory

This section provides a brief review of perturbation theory as presented by Stevens [5], which is used in the present study to model and explain the effect of vocal tract constrictions on formant frequencies. A more complete exposition of perturbation theory can be found in the reference provided. Only the details that are important in the derivation of Eq (16) are presented here.

Standing wave patterns in a completely uniform vocal tract produce a pressure value at *x*, a location along the length of the vocal tract from the lips (*x* = 0) to the glottis, of the following form:

$$\begin{array}{c} p_{n}\left(x\right)=P_{m}sin\frac{2\pi F_{n}x}{c} \end{array}$$ (11)

where *P*_*m*_ is the maximum pressure in the vocal tract, *F*_*n*_ is the *n* ^*th*^ natural frequency of the vocal tract and *c* is the speed of sound inside the vocal tract. The volume velocity profile also has a sinusoidal shape, following:

$$\begin{array}{c} U_{n}\left(x\right)=jP_{m}\frac{A}{\rho c}cos\frac{2\pi F_{n}x}{c} \end{array}$$ (12)

where *A* is the cross-sectional area of the uniform tube under consideration, and *ρ* is the ambient density of air.

When an initially-uniform tube is subsequently constricted at some location along its length, the amount of stored energy in the system (*W*) is changed as a function of both the potential (*V*) and kinetic energy (*T*) in the system:

$$\begin{array}{c} \Delta W_{n}=\Delta V_{n}+\Delta T_{n} \end{array}$$ (13)

where *n* is an integer label referring to a specific natural frequency. Changes in stored energy cause a shift in natural frequencies *dF*_*n*_ = −Δ*W*_*n*_ *F*_*n*_/*W*_*n*_, where *W*_*n*_ is the total energy in the system. The equation is the well-known Boltzmann-Ehrenfest Theorem, which states that the resonant frequencies of an oscillator are proportional to the amount of energy in the system. Changes in stored potential energy can be expressed as

$$\begin{array}{c} \Delta V_{n}=\frac{1}{4}{|p_{n}\left(x\right)|}^{2}\frac{\Delta l\Delta A}{\rho c^{2}} \end{array}$$ (14)

and changes in stored kinetic energy can be expressed as

$$\begin{array}{c} \Delta T_{n}=-\frac{1}{4}{|U_{n}\left(x\right)|}^{2}\frac{\rho \Delta l\Delta A}{A^{2}} \end{array}$$ (15)

for small changes in the cross-sectional area of the tube, Δ*A*, at location *x* and over a short length of the tube, Δ*l*. This model assumes that the pressure and volume velocity profiles are not substantially altered by small perturbations of the vocal tract area or length. It has been repeatedly shown that this assumption is reasonable for relatively unconstricted vocal tract shapes, as in vowels (e.g., as described by Mrayati [32]). By extending this assumption to several adjacent short area perturbations, the acoustic consequences of vocal tract constrictions with a longer spatial extent can be modeled by summing in the following way:

$$\begin{array}{c} \Delta W_{n}=\sum_{a=-t/2}^{t/2}\Delta V_{n}\left(x_{a}\right)+\Delta T_{n}\left(x_{a}\right) \end{array}$$ (16)

where *x*_*a*_ = *x*_0_+*a*Δ*l*, *x* is perturbation location variable defined with respect to Eqs (11) and (12), and *x*_0_ is the spatial center of the long constriction. Thus, this relatively long constriction is composed of *t* adjacent short perturbations, and the value *t*Δ*l* is the overall length of the constriction. It may seem necessary to employ more sophisticated models for calculating changes to the pressure and volume velocity profiles (e.g., as utilized by Story [33]). However, it will be shown empirically later in the present paper that perturbation theory produces highly meaningful results when extended in this way, at least for the relatively simple constriction shapes considered here.

### Data for Estimation Experiments

To conduct experiments regarding the estimation of vocal tract length from formant frequencies, two data sets were gathered: one composed of simulated speech data from the multi-tube acoustic model, and one composed of real human speech data obtained from rtMRI. The sections, Simulated Speech Data and Human Speech Data, describe the details of how those data sets were gathered. Consideration of resonances was limited to the first four formant frequencies in all cases, due to the difficulty associated with estimating higher formants from human speech data.

Having two data sets also provides an opportunity to analyze whether the modeling assumptions behind the proposed estimator framework, which incorporates the previous and proposed estimators, are reasonable. The synthetic data maintain the assumptions of losslessness and of an idealized radiation impedance, but not of uniformity of the area function. The human speech data, by contrast, are likely to violate all of these assumptions to some degree. Thus, the differential accuracy of the proposed estimator on the two data sets should provide an indication of how much estimator error can be attributed to normal articulation of the vocal tract versus modeling assumptions behind the estimator, though one must keep in mind that the human speech data may also contain measurement error.

#### Ethics Statement

All relevant research in the present study was approved by the Institutional Review Board at the University of Southern California (UPIRB: https://oprs.usc.edu/upirb/). All participants provided written informed consent prior to participation, and participant records and information were anonymized and de-identified prior to analysis.

#### Simulated Speech Data

Simulated formant frequencies were gathered from a set of randomly-generated vocal tract area functions. Area functions were parameterized using the spatial discrete Fourier transform of vocal tract area functions developed by Schroeder [17] and Mermelstein [16], and later utilized by Iskarous [34]. This parameterization is based on representing an arbitrary area function as a linear combination of spatial sinusoids defined along the vocal tract’s length. In this work, a spatial half-cosine and its first five integer harmonics were used as the basis for representation. Specifically, at *x*, a location along the length of the vocal tract from the glottis (*x* = 0) to the lips, the area function’s deviation from a uniform shape is:

$$\begin{array}{c} \delta A\left(x\right)=A_{0}\sum_{n=1}^{6}a_{n}cos\left(\frac{\pi nx}{L}\right), \end{array}$$ (17)

where *n* is the integer label of the sinusoidal harmonic component and *A*_0_ is the cross-sectional area of a uniform vocal tract in cm^2^. In this case, *A*_0_ = *π*, corresponding to a midsagittal distance of 2 cm. The area function at this location is then,

$$\begin{array}{c} A\left(x\right)=A_{0}+\delta A\left(x\right) \end{array}$$ (18)

where coefficients *a*_1…6_ determine the contribution of each sinusoid to the overall shape. The harmonic series of six sinusoidal components can also be regarded as three components that are symmetric along the vocal tract (i.e., the even harmonics), and three that are anti-symmetric (i.e., the odd harmonics).

Although originally developed to represent known area functions, this area function parameterization was used in the present study to generate a total of 3,200 area functions for six simulated “speakers”, each with a different vocal tract length, ranging from 14 to 19 cm at steps of 1 cm. Area functions were generated by random selection of the coefficients, *a*_1…6_, from a uniform distribution with range (-1,1). Random selection of coefficients in this way produces area functions with very small or negative values—here defined as areas less than 0.1 cm^2^—approximately 30% of the time, at locations distributed along the entire length of the area function. During the generation process, any area functions displaying an area less than this pre-specified amount were discarded. This process of random coefficient generation and checking for sub-threshold values was repeated for a given simulated speaker until 3,200 area functions were compiled. Repeating this for all six speakers amounted to a total of 19,200 total area functions in the data set.

**Fig 1:**
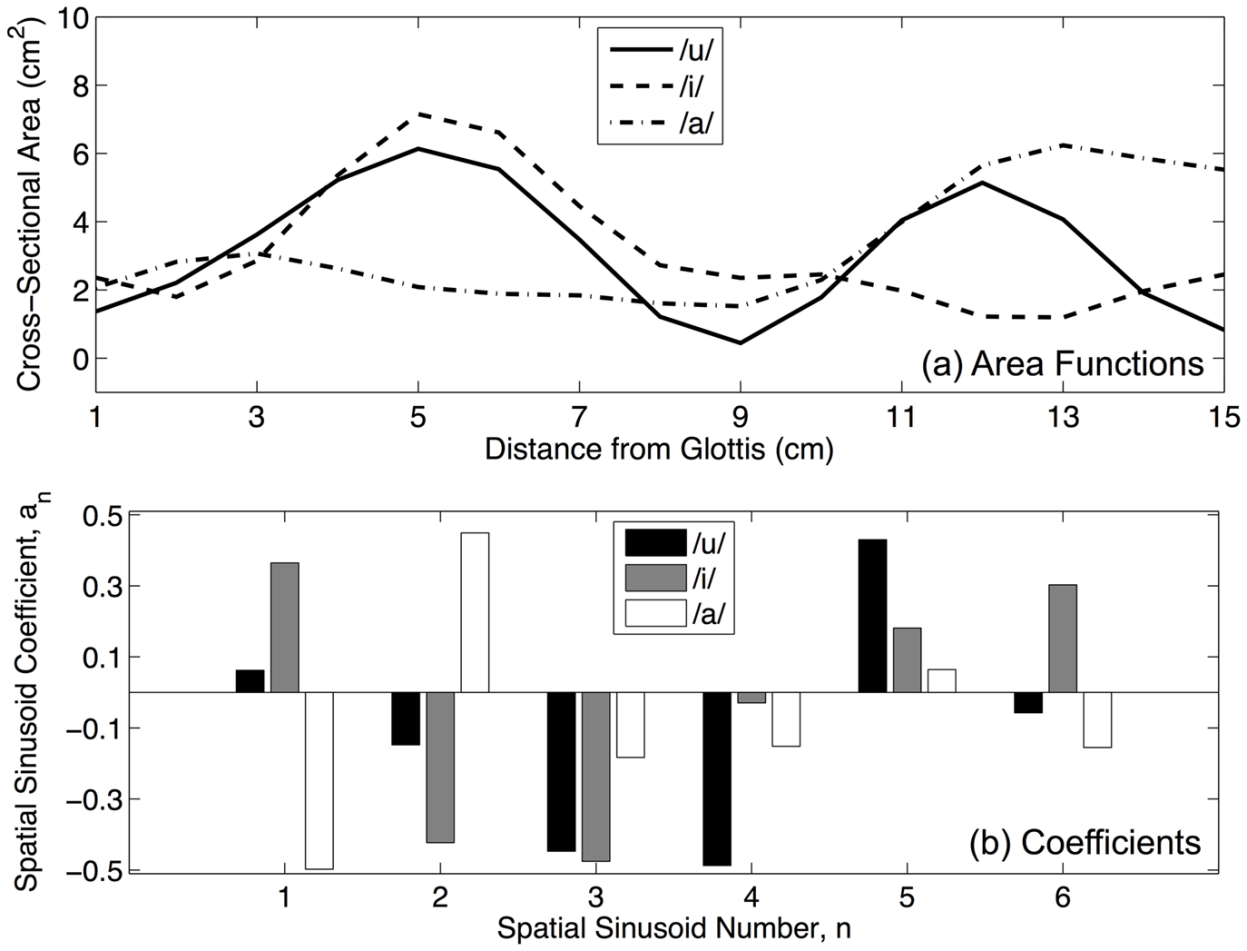
Three randomly-generated area functions (a) from the simulated speech data set and their corresponding coefficient values (b) that define their shape according to Eqs (17) and (18). These example area functions were found to be closest, in terms of RMS error, to the area functions presented by Wood [35] for the English vowels /u/, /i/ and /a/.

Using this area function generation procedure, a wide variety of vocal tract shapes can be generated automatically. Fig 1 shows three area functions extracted from the data which were found to be closest, in terms of mean squared error, to the area functions presented by Wood [35] for the English vowels /u/, /i/ and /a/. The overall range of cross-sectional area in the entire data set was 0.1 cm^2^—11.3 cm^2^, corresponding to an overall range of midsagittal distances of 0.36—3.79 cm. Each whole-centimeter location along the length of the area function displayed a standard deviation of approximately 1.5 across the entire data set, indicating that no single location exhibited diminished area variation compared to the others.

Formant frequencies were generated from these area functions using the multi-tube model. This necessitated the conversion of continuous area functions into vectors of finite area measurements, with each measurement representing the cross-sectional area of one tube in the model. All tubes were considered to have a constant length of 0.25 cm, making the number of tubes change with different overall vocal tract lengths, from 56 (14 cm) to 76 (19 cm).

#### Human Speech Data

Human speech data were collected from five native speakers of American English (two male, three female). Each subject spoke five sentences from the MOCHA-TIMIT corpus (see Table 1). Data were acquired at Los Angeles County Hospital on a Signa Excite HD 1.5T scanner (GE Healthcare, Waukesha WI). A custom 4-channel upper airway receiver coil array was used for RF signal reception, with two anterior coil elements and two coil elements posterior to the head and neck. A 13-interleaf spiral gradient echo pulse sequence was used (*T*_*R*_ = 6.164 msec, FOV = 200 × 200 mm, flip angle = 15 degrees, receiver bandwidth = ±125 kHz). Scan plane localization of the 5 mm mid-sagittal slice was performed using RTHawk (HeartVista, Inc., Los Altos, CA), a custom real-time imaging platform [36]. Images were reconstructed at a rate of 23.33 frames/second. Image resolution after reconstruction was 68 × 68 pixels at 2.9 × 2.9 mm pixel width. More details about MRI acquisition of vocal tract images can be found in [37–42]. Synchronous audio recordings of the subjects’ speech were also acquired using an optical microphone. Audio were subsequently denoised according to the protocol described by Bresch [43]. This denoising technique promises nearly 30dB noise suppression during speech and, in general, minimal errors are expected in terms of spectral distortion as a result of applying this method. However, due to the nature of the recording environment inside the scanner bore, one may expect some reverberation and background noise caused by the cryogen pump and ventilation system.

**Table 1:** Sentences used as stimuli for eliciting the analyzed utterances. All subjects spoke these same five sentences.

1. This was easy for us.
2. Is this seesaw safe?
3. Those thieves stole thirty jewels.
4. Jane may earn more money by working hard.
5. She is thinner than I am.

Audio was analyzed using Praat [44] for both formant and pitch tracking. Formant analysis in Praat is based on linear predictive coding (LPC) analysis, which attempts to separate vocal tract filter characteristics (e.g., formants) from the glottal source characteristics. Formant tracking was configured to find six formants in the range 0–5500Hz with a window length of 25ms. These settings provide superfluous and potentially spurious formant tracks, but also provided the most accurate tracking of the formant frequencies of interest when overlaid on a spectrogram of the same utterance and visually inspected. Formants of interest were identified by this overlay procedure, and irrelevant formant values were discarded. Pitch tracking was configured to find a pitch value in the range 75–500Hz using the autocorrelation method with a window length of 10 ms. Pitch measurements were used to remove non-sonorant sounds from further analysis. Formant analysis frames were eliminated from further consideration if a reliable pitch could not be found by the pitch tracking algorithm in the temporally nearest pitch analysis frame. In total, this resulted in 3,870 frames of data, or approximately 775 frames per subject.

In order to measure vocal tract length for each subject, vocal tract images for each subject were extracted from the rtMRI video sequences at times corresponding to the retained formant measurement frames. From this subset of video frames, an overall mean image was formed for each subject by taking the pixel-wise mean intensity value across all the images in the subset. This overall mean image is a representation of the average vocal tract posture assumed by the subject during production of the formants in the data set. However, calculating a mean image over such a large number of images (approximately 200 frames per speaker) results in a mean image which is blurred and unsuitable for finding vocal tract outlines/midlines. Therefore, one additional mean image was calculated for each subject from the ten images that were closest to the overall mean image in terms of having the smallest sum of squared pixel intensity differences across all pixels in the image plane. From this final, unblurred mean image, the vocal tract outlines were traced.

**Fig 2:**
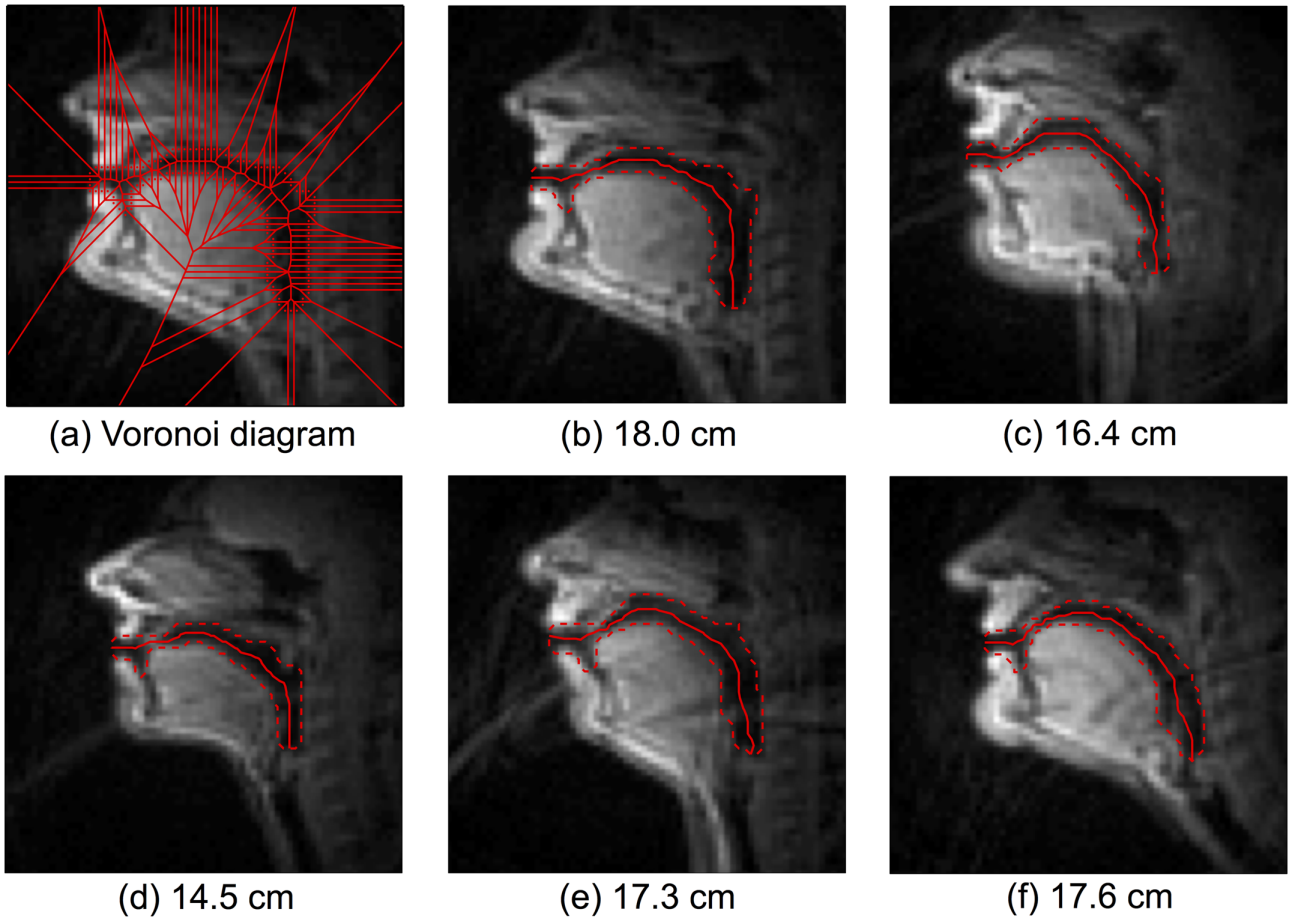
Midsagittal rtMR image of subject al1 (a) with vocal tract outlines and Voronoi diagram overlaid. Mean images of the five subjects (b–f), a representation of the average vocal tract posture assumed by the subject during production of the formants in the data set. Vocal tract outlines are overlaid (dashed lines) with the vocal tract midlines (solid lines) calculated from those outlines using Voronoi skeletons. The length of these midlines was used as the measure of vocal tract length for each subject. The specific values of vocal tract length for each subject were as follows: b: 18.0 cm, c: 16.4 cm, d: 14.5 cm, e: 17.3 cm, f: 17.6 cm

Vocal tract outlines were found using Canny edge detection [45] with manual linking and correction. For all five subjects, this procedure resulted in an outer vocal tract contour following the contour of the upper lip, alveolar ridge, hard palate, velum and posterior pharyngeal wall. The teeth were excluded from outlines, as they are not visible in MRI and typically ignored, in any event, with respect to vocal tract midline determination and length measurements. The lower vocal tract contour followed the lower lip and the tongue from its tip to below its attachment point of the epiglottis. The vocal tract endpoints were identified by inspection, with the lower point being the upper edge of the false vocal folds. The Voronoi skeleton [46] was subsequently calculated from these outlines. Voronoi vertices were selected that were equidistant from vocal tract outlines, forming the shortest path between the end vertices. The vocal tract outlines and resulting midlines for all five subjects can be seen in Fig 2. The mean vocal tract length across these five subjects was 16.8 cm.

The vocal tract outlines extend to the upper edge of the false vocal folds in the caudal direction, and not down to the vocal folds proper. The false vocal folds represent a consistently identifiable anatomical landmark in the images, that extend across the entire sagittal length of the larynx in the anterior-posterior direction. To account for the distance between the upper edge of the false vocal folds and the glottis, 10% was added to all length measurements. Kitzing [47] made detailed measurements of the adult male laryngeal ventricle during phonation and found that, at a typical male phonation frequency of 125 Hz, the length of this structure was 16.6 mm. This length is approximately 9% of average male vocal tract length, which was rounded up to 10% due to our observation that both males in our data set were slightly above the average in terms of body size and height.

### Length Estimation Experiments

Using both the simulated and human speech data that were collected, a series of experiments were carried out to evaluate the performance of several vocal tract length estimators described in the Vocal Tract Length Estimation Framework section. The evaluated estimators included Fitch’s [22] Frequency Dispersion (Eq (6)), Wakita’s [21] proposal of using the highest formant (i.e., F4) and mean of the two highest formants (i.e., F3 and F4), Kirlin’s [19] maximum likelihood estimator (Eq (7)), and the proposed regression-based method with coefficients determined as in Eq (9). The precise specifications of these estimators are given in Tables 2 and 3.

Experiments were conducted using a repeated resampling procedure with heldout data, with experiments conducted separately on the synthetic and human speech data sets. Non-overlapping subsets of the full data sets were utilized for training and testing the estimators. Half of the data, including formant frequency vectors with corresponding vocal tract lengths, were randomly assigned to a training set, used to obtain the regression coefficients. The other half of the data were assigned to a test set, used to evaluate the accuracy of the estimates. This procedure of random assignment, model fitting and evaluation was repeated 1000 times. Resampling the data in this way should provide a more robust estimate of the overall accuracy by examining the mean accuracy over all repetitions, as well as a better indication of how the results will generalize to other data sets. Resampling also affords an estimate of the stability of the model coefficients by examining their standard deviation over all repetitions.

### Formant Sensitivity Experiments

Wakita’s [21] idea to use higher formants, alone or in combination, to predict vocal tract length was based on the suggestion that higher formants are less sensitive to speech articulation. The *sensitivity* of a formant, in this context, refers to the amount of variation in that formant’s observed frequency, expressed as a proportion of its expected frequency during a neutral area function shape. Decreased sensitivity of certain formants across a variety of area function shapes would make those formants better predictors of vocal tract length. Formants frequencies are always determined, in large part, by some combination of vocal tract length and area function shape, which implies that formants which are less affected by changes in area function shape would provide relatively more reliable information about length alone. It was observed that the design of the proposed, data-driven estimator, as shown in Tables 2 and 3, places comparatively more weight on higher formants. This finding is consistent with the idea that higher formants are more reliable as features, perhaps because they are less sensitive. Indeed, higher formants appear to vary less across the vocal tract configurations contained in the collected data sets. Table 4 shows that the normalized standard deviation of formant frequencies decreases monotonically as higher formants are considered. Further empirical verification for this suggestion was sought.

**Table 2:** Estimation accuracies of several estimators on simulated speech data in terms of RMS error, along with the parameters that define the estimator. For estimators with no parameter *β*_0_, the coefficients *β*_1_…*β*_4_ correspond to those in Eq (4). For estimators with all five parameters listed, those parameters correspond to those in Eq (20). Note that the coefficient values for the maximum likelihood estimator, the proposed estimator, the maximum *a posteriori* estimator and the extended proposed estimator are mean values across all resamplings. Standard deviations of the coefficients *β*_1…4_ for all estimators across all resamplings was < 0.005, and standard deviations of the coefficients *β*_0_ was < 5.

Estimator	*β*_0_	*β*_1_	*β*_2_	*β*_3_	*β*_4_	RMSE (cm)
Freq. Dispersion		−0.167	0.000	0.000	1.167	1.692
*F*_4_ only		0.000	0.000	0.000	1.000	1.206
Mean(*F*_3_, *F*_4_)		0.000	0.000	0.500	0.500	1.194
Max. Likelihood		0.083	0.122	0.192	0.604	0.683
Proposed		0.089	0.102	0.121	0.669	0.631
Max. a Post.	144	0.062	0.089	0.147	0.434	0.671
Proposed—Ext.	52	0.078	0.099	0.101	0.609	0.608

**Table 3:** Estimation accuracies of several estimators on human speech data in terms of RMS error, along with the parameters that define the estimator. For estimators with no parameter *β*_0_, the coefficients *β*_1_…*β*_4_ correspond to those in Eq (4). For estimators with all five parameters listed, those parameters correspond to those in Eq (20). Note that the coefficient values for the maximum likelihood estimator, the proposed estimator, the maximum *a posteriori* estimator and the extended proposed estimator are mean values across all resamplings. Standard deviations of the coefficients *β*_1…4_ for all estimators across all resamplings was < 0.015, and standard deviations of the coefficients *β*_0_ was < 10.

Estimator	*β*_0_	*β*_1_	*β*_2_	*β*_3_	*β*_4_	RMSE (cm)
Freq. Dispersion		−0.167	0.000	0.000	1.167	4.125
*F*_4_ only		0.000	0.000	0.000	1.000	2.621
Mean(*F*_3_, *F*_4_)		0.000	0.000	0.500	0.500	2.067
Max. Likelihood		0.041	0.149	0.441	0.369	1.556
Proposed		0.022	0.136	0.254	0.637	1.277
Max. a Post.	253	0.021	0.078	0.228	0.193	0.946
Proposed—Ext.	229	0.030	0.082	0.124	0.354	0.839

**Table 4:** Empirical comparison of the sensitivity of formant frequencies to speech articulation in the simulated and human speech data sets. The measure presented is normalized standard deviation: $\frac{\sigma_{F_{n}}}{2n-1}$, where *n* is the formant number. Lower numbers indicate less sensitivity to articulation. Note that sensitivity decreases as higher formants are considered.

Formant #	Simulated Speech Data	Human Speech Data
1	104.0	198.0
2	98.3	102.9
3	97.2	74.7
4	65.1	55.0

Experiments were performed using a simulated vocal tract 17 cm in length, closed at one end, with uniform cross-sectional area of *π* cm^2^. Vocal tract constrictions were represented as uniformly narrowed sections with an area of *π* − 0.5 cm^2^. The lengths of these constricted sections ranged from 0.25 to 8.5 cm (i.e., 1.5 to 50% of total vocal tract length) in 0.25 cm steps. Constrictions were generated such that the central point of their spatial extent—the constriction location—was placed at every possible location along the length of the tract. Note that, although a single value of constriction degree was used, according to perturbation theory (see Eqs (13) and (14)), changing the constriction degree should only act as a scaling factor on the sensitivity of all formants, and therefore not affect comparisons of sensitivity across formants. Formant frequencies were calculated for vocal tracts of the specified parameters using both multi-tube and perturbation modeling techniques described in the section Acoustic Modeling. Perturbation theory would suffice for these sensitivity experiments, and can also provide deeper insights into the reasons for the formant sensitivity differences, as will be discussed later. However, it is not immediately clear that the assumptions made in extending the theory from a single constriction to multiple ones (see Eq (16)) are reasonable, nor is it clear whether a perturbation of the size assumed here is reasonably small. Concern over these assumptions compelled additional simulations using the multi-tube model, to provide corroborating evidence for their adoption. It will be shown that the simulation results using either method match very closely, indicating that these assumptions are, indeed, reasonable.

## Results and Discussion

### Length Estimation Experiments

Accuracies of various estimators on the simulated data set are shown in Table 2. Results on human speech data are shown in Table 3. Accuracies are presented in terms of the root mean squared error (RMS error) across all test data. The specific estimator coefficients, corresponding to those in Eq (4), are also listed. RMS error of the proposed estimator on simulated data was 0.631 cm, which is 3.82% of the mean vocal tract length in the data set. The proposed estimator achieved an RMS error of 1.277 cm on the human speech data, which is 7.60% of the mean vocal tract length in that data set.

### Formant Sensitivity Experiments

**Fig 3:**
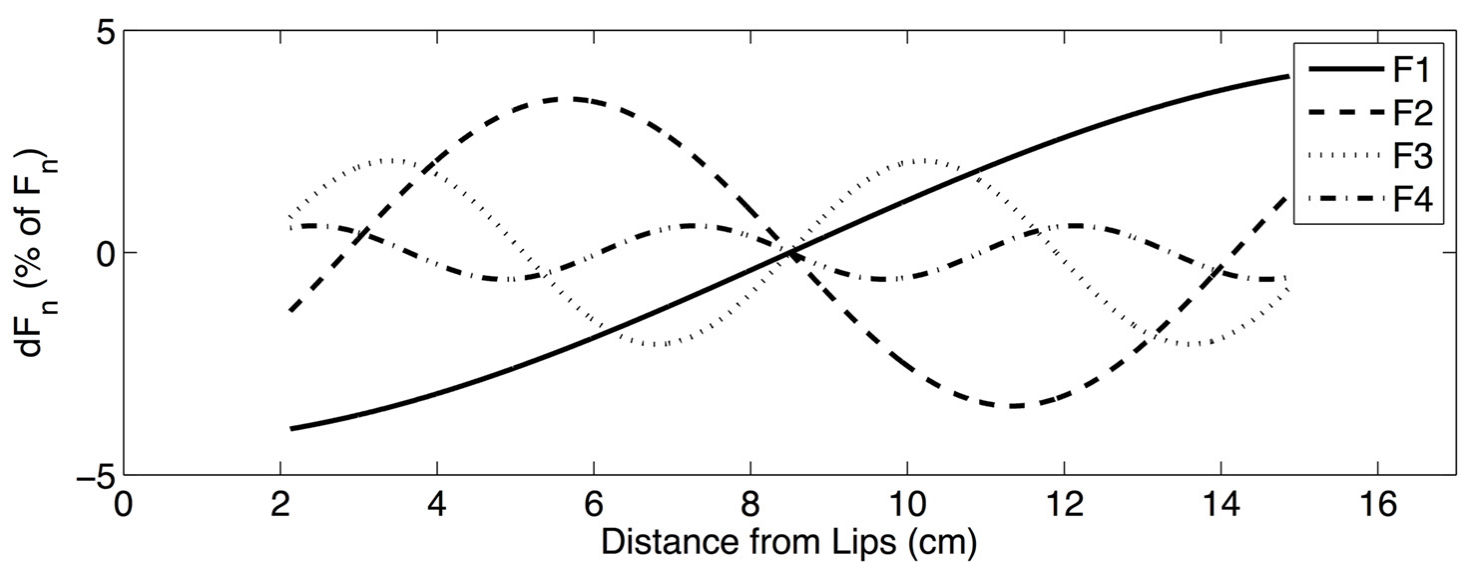
Sensitivity functions predicted by perturbation theory, showing the sensitivity of the first four formants to a vocal tract constriction whose center is placed at all possible locations along the length of the vocal tract. This example uses a vocal tract constriction of uniform area, with a length that is 25% of total vocal tract length. Note that the range of the sensitivity functions decreases as higher formants are considered. The range of sensitivity functions can be used as a measure of formant sensitivity to constrictions of a given length, regardless of their location, as in the sensitivity range functions shown in Figs 4 and 5.

**Fig 4:**
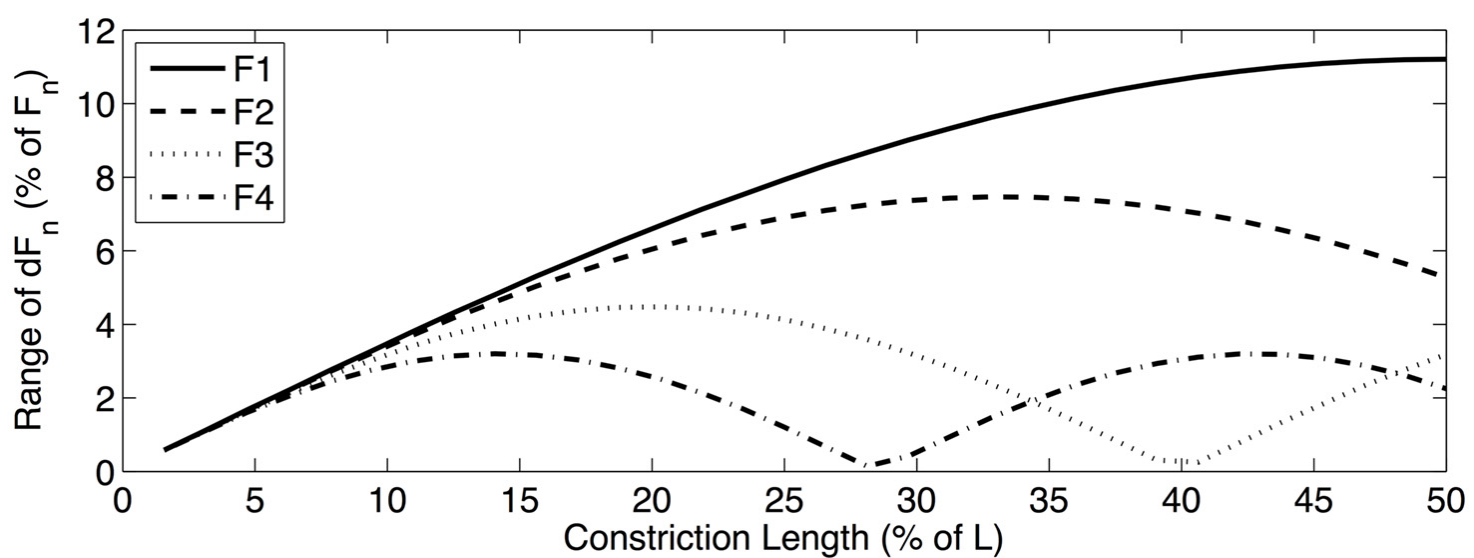
Sensitivity range functions predicted by perturbation theory, showing the range of sensitivity of the first four formants to vocal tract constrictions of different lengths, regardless of their location. This example uses uniform vocal tract constrictions with lengths varying from 0 to 50% of total vocal tract length. Note that, in general, the sensitivity range decreases as higher formants are considered, although there are some exceptions to this general trend. When constrictions are very small (i.e., less than 5% of total vocal tract length), there is not much difference between the sensitivity ranges of different formants. There are also some exceptions to the decreasing sensitivity trend, as can be seen when constrictions are between approximately 34% and 48% of total vocal tract length. In that range, F4 is more sensitive than F3.

**Fig 5:**
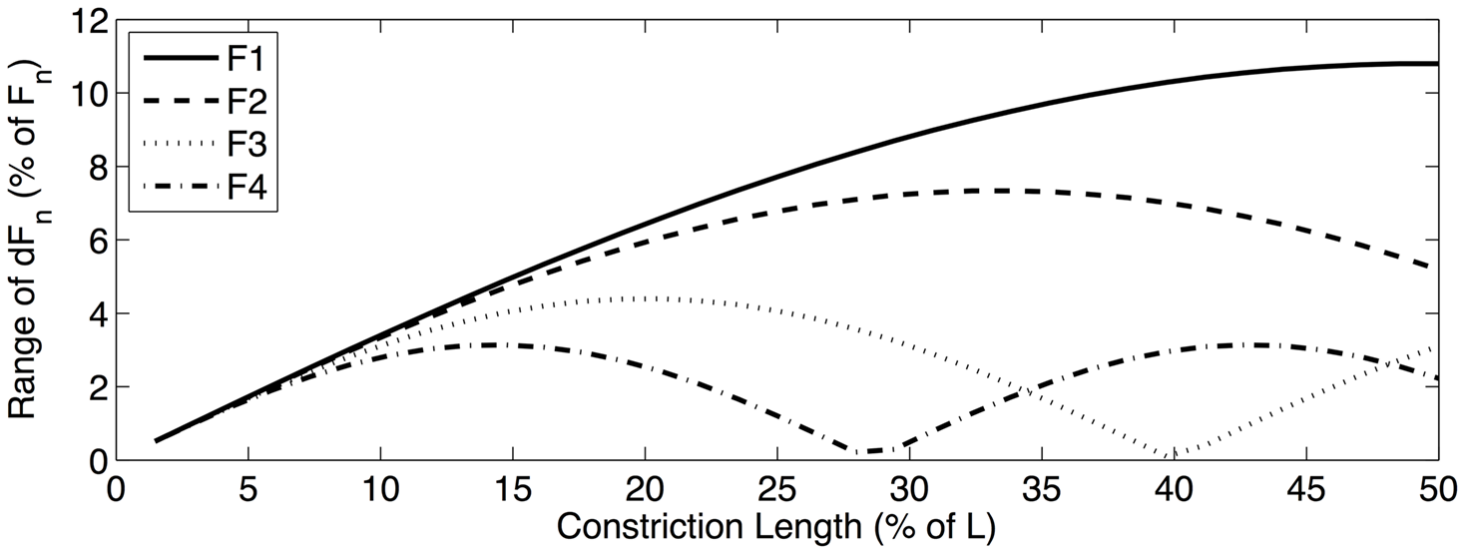
Sensitivity range functions predicted by the multitube model, showing the range of sensitivity of the first four formants to vocal tract constrictions of different lengths, regardless of their location. This example uses uniform vocal tract constrictions with lengths varying from 0 to 50% of total vocal tract length. Note the striking similarity to the results predicted by perturbation theory, as shown in Fig 4, which provides corroborating evidence that the proposed assumptions behind and extensions to perturbation theory are reasonable.

Results of the formant sensitivity experiments can be summarized in two ways. First, formant sensitivity can be shown as a function of the constriction location along the length of the vocal tract. In order to effectively visualize these *sensitivity functions*, a particular constriction length must be chosen. Fig 3 shows the sensitivity functions for the first four formants when a vocal tract constriction that is 25% of the total vocal tract length is chosen. The range of these sensitivity functions can also be calculated and used as an indication of overall formant sensitivity as a function of constriction length, without regard to the specific location of the constriction. Fig 4 shows these *sensitivity range functions*, as calculated using perturbation theory, across all constriction lengths considered in the formant sensitivity experiments. Fig 5 shows the sensitivity range functions calculated using the multitube model. The fact that Figs 4 and 5 are strikingly consistent (though not identical) can be taken as evidence that perturbation theory can meaningfully be extended in a way consistent with Eq (16).

### Optimal Estimation of Vocal Tract Length

The proposed vocal tract length estimator displays the lowest estimation error compared to previously proposed estimators on both simulated and human speech data. This result is perhaps not surprising, given that the proposed estimator is optimal, in the least-squares sense, among estimators belonging to the framework developed here on the data sets used in the present paper. It should be noted, however, that this optimality is only guaranteed over the data used to train the estimator, although the heldout data validation scheme employed here lends confidence that the proposed estimator’s performance is generalizable. Kirlin’s [19] maximum likelihood estimator—the only other estimator developed in a data-driven fashion—displays the second best performance on both data sets, and is very similar in terms of accuracy on the simulated data set. The maximum likelihood estimator has several conceptual differences from the proposed estimator, which may result in the differences in accuracy between the two. In particular, the maximum likelihood estimator is derived from a full probabilistic treatment of vocal tract length estimation, which necessitates additional simplifying assumptions that the proposed estimator does not require. Most notable among these assumptions is that individual formants will be Gaussian-distributed about their presumed center frequency, which is likely to be an oversimplification. The remaining estimators display substantially lower performance on the data sets used in the present study. The rank order of methods by accuracy is precisely the same on both data sets.

The proposed estimator, whether it is trained on simulated or human speech data, exhibits coefficients that strictly increase in value as higher formants are considered. This shows that all formants provide some information about vocal tract length, and it strongly implies that higher formants provide increasingly reliable information. As such, the design of the proposed estimator is consistent with the idea that higher formants are less sensitive to speech articulation. Further empirical evidence and a theoretical justification for this idea are presented in the discussion section entitled “Formant Sensitivity Differences”. Note, too, that the maximum likelihood estimator also takes on coefficients that generally increase with formant number. This increase is monotonic when the estimator is trained on simulated speech data, but not strictly monotonic when trained on the human speech data.

Accuracies on human speech data are generally lower than accuracies on simulated data. Using the proposed estimator, for example, the difference in RMSE is approximately 0.6 cm, or between 3 and 4% of average vocal tract length. This difference highlights errors resulting from the simplifying assumptions made in developing the previous and proposed estimators, and in developing the general estimation framework of Eq (4). Those assumptions include idealized geometry and radiation impedance, and lack of loss, all of which are also true of the simulated speech data set, but not the human speech data. Errors resulting from these assumptions can be addressed by incorporating more physical knowledge into the estimator framework, with the goal of eliminating certain assumptions altogether. Such changes will be most feasible for assumptions that do not depend critically on knowledge of speech articulation (e.g., lossy sidewalls). Assumptions that do depend on articulation knowledge (e.g., the radiation impedance) may still be refined, however, by modifying or weakening the assumptions. Other obvious sources of error for estimates on human speech data include measurement noise introduced during formant tracking or when measuring vocal tract length. Although Praat’s LPC-based formant analysis produced satisfactory results for present purposes, it is likely that algorithms tailored toward the unique recording characteristics of real-MRI audio could improve the accuracy of formant tracking. Similarly, higher resolution and higher contrast vocal tract image would allow for even more detailed, and potentially more accurate measurements of vocal tract shape and length. The human speech data set is also considerably smaller than the simulated data set, and increasing the amount of human speech data would likely lead to small improvements.

Eliminating any remaining estimation error may require a more complex model than stated in Eq (4). Model complexity can be increased in the most straightforward way by either adding model parameters or adding input features. One possible extension of the model, that of adding parameters, is explored in the discussion section entitled “Framework Extension”. Input features that might be added to the model include acoustical features, such as formant bandwidths. However, it seems likely that some knowledge of speech articulation must be incorporated to make this problem well-posed, because changes in the area function are a large—even dominant [8]—source of formant variation. Even if a purely acoustical method of estimation is desired, some knowledge of the area function could still be utilized if that knowledge was obtained through acoustic-to-articulatory inversion, although inverted features would likely eliminate the possibility of a closed-form estimator.

### Formant Sensitivity Differences

Several empirical results in this study reinforce the idea that higher formants are relatively less sensitive to speech articulation as compared to lower formants. Direct examination of formant frequencies in the data sets presented here reveals that each formant varies proportionally less than the formant just below it. Moreover, the final design of the proposed estimator indicates that higher formants provide more reliable information about vocal tract length because their observed frequencies are determined relatively less by area function shape. This evidence is further corroborated by formant sensitivity experiments presented here, which indicate that both perturbation theory and the widely-used multitube model predict similar effects. Formant sensitivity decreases as higher formants are considered, in general. However, these sensitivity differences vary as a function of constriction length. For constrictions between 0% and approximately 33% of the vocal tract length, this characterization is accurate, with the reduction in sensitivity becoming most dramatic for constrictions just below 30% of vocal tract length. On the other hand, this effect is very small for constrictions less than 10% of vocal tract length. For constrictions approximately between 33% and 48% of overall vocal tract length, this effect still holds for *F*_1_ and *F*_2_, but there is a reversal for *F*_3_ and *F*_4_. Using the perturbation theory model, deeper insights into the reasons for this effect can also be found.

**Fig 6:**
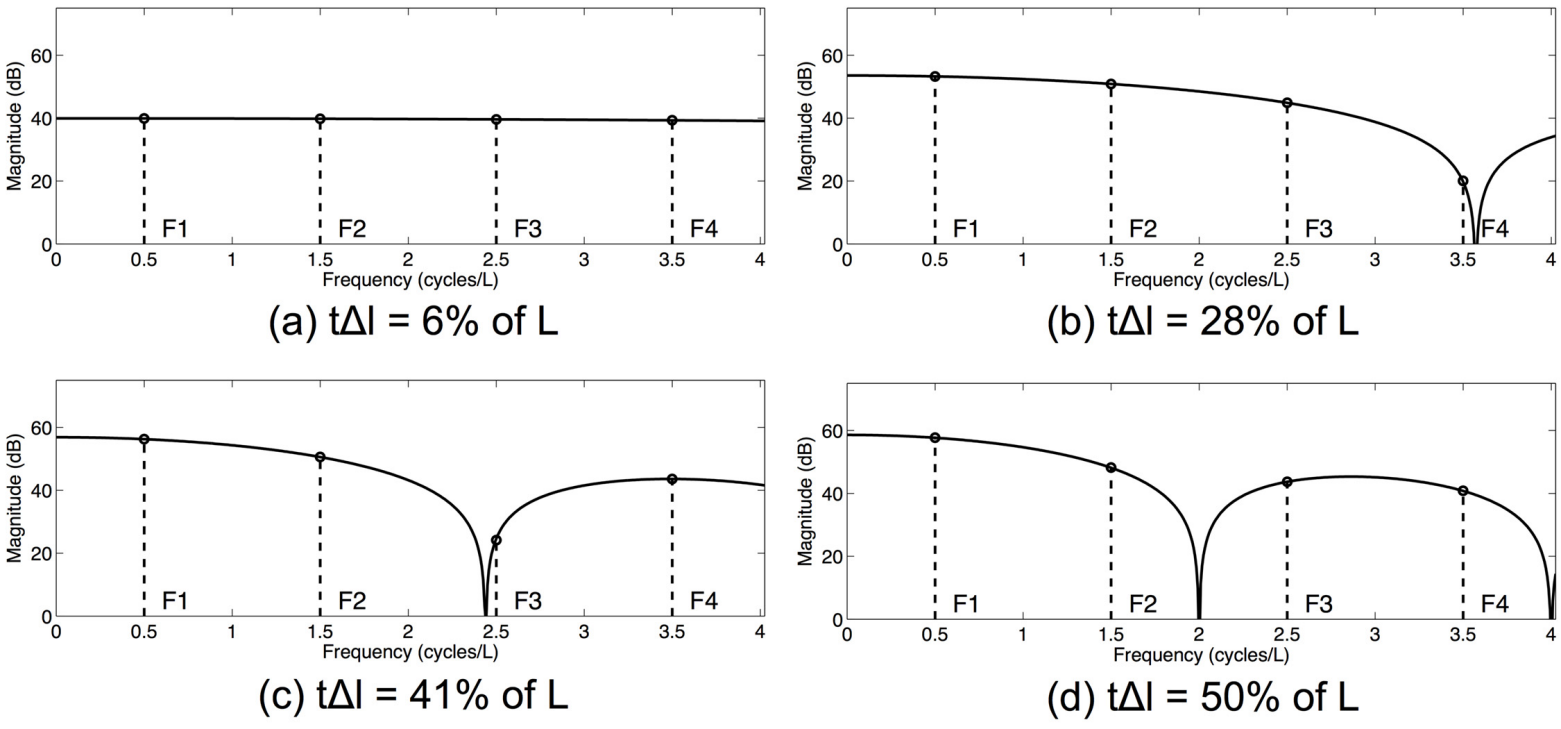
Frequency response of the filter specified by Eq (19), which reflects an interpretation of vocal tract constrictions, presented in Eq (16), as a summation over some section of the sensitivity function defined for single, short perturbations. The transfer function depends on the length of the constriction, *t*Δ*l*, which is why the frequency response for different constriction lengths are shown. Overlaid are lines indicating the fundamental frequencies of single-perturbation sensitivity functions corresponding to the first four formant frequencies. When constriction length is long (d, *t*Δ*l* = 50% of L), magnitude of the frequency response generally decreases as the spatial frequency of the sensitivity function increases, corresponding to the overall reduction in the sensitivity of higher formants. For very short constrictions (a; *t*Δ*l* = 6% of L), this sensitivity difference is marginal. It is also possible to get very dramatic reduction of sensitivity for higher formants (b; *t*Δ*l* = 28% of L), or to maximally attenuate the sensitivity of non-highest formant (c; *t*Δ*l* = 41% of L). These results are highly consistent with the sensitivity range functions from the formant sensitivity experiments, as presented in Figs 4 and 5.

One interpretation of this effect can be stated precisely by observing that Eq (16), which represents the sensitivity of some formant frequency to a relatively long constriction, can be interpreted as a summation over some section of the sensitivity function defined for single, short perturbations. Applying this summation across all constriction locations along the vocal tract is equivalent to applying a finite impulse response filter to the single-perturbation sensitivity function, where the filter, which is non-causal in the spatial domain, can be represented with numerator coefficients equal to one. In particular, the output of the filter is defined as:

$$\begin{array}{c} Y\left[x\right]=S\left[x+\frac{t\Delta l}{2}\right]+\ldots +S\left[x+\Delta l\right]+S\left[x\right]+S\left[x-\Delta l\right]+\ldots +S\left[x-\frac{t\Delta l}{2}\right], \end{array}$$ (19)

where *x* is the spatial location along the vocal tract, *S*[*x*] are the input samples of the single-perturbation sensitivity function, and *Y*[*x*] is the filtered long-perturbation sensitivity function. The transfer function of this filter depends heavily on the length of the constriction, *t*Δ*l*. Fig 6 shows the frequency response of the filter for a variety of constriction lengths. Overlaid are lines indicating the fundamental frequencies of single-perturbation sensitivity functions corresponding to the first four formant frequencies. These frequencies can be determined by examining Eqs (11) and (12), which show that the pressure and volume velocity profiles vary according to sine and cosine of the position along the length of the vocal tract. If perturbations of a constant area along their length are considered, as before, then the pressure and volume velocity profiles will be sinusoids with spatial frequencies (i.e., wavenumber) equal to *F*_*n*_/*c*. The short-perturbation sensitivity function is formed by the sum of these two sinusoids after they have been full-wave rectified, resulting in a fundamental frequency of 2*F*_*n*_/*c*.

Examining the frequency response for the longest constriction length (d), it is possible to see that the magnitude of the frequency response generally decreases as the spatial frequency of the sensitivity function increases, corresponding to the overall reduction in the sensitivity of higher formants. For constrictions that are quite short (a), this sensitivity difference is marginal. Depending on the specific length of the constriction, it is possible to get very dramatic reduction of sensitivity for higher formants (b). In certain cases, however, it is possible to most attenuate the sensitivity of non-highest formant (c). These results are highly consistent with the sensitivity range functions from the formant sensitivity experiments.

### Framework Extension

Perhaps the most obvious way to extend the basic estimation framework of Eq (4) is to add a constant offset term, *β*_0_, which would make this a full multiple linear regression model, of the following form:

$$\begin{array}{c} \hat{\Phi }=\beta_{0}+\frac{\beta_{1}F_{1}}{1}+\frac{\beta_{2}F_{2}}{3}+\frac{\beta_{3}F_{3}}{5}+\cdots +\frac{\beta_{m}F_{m}}{2m-1}. \end{array}$$ (20)

Kirlin [19] showed that the addition of an offset term can be motivated in a probabilistic way. Specifically, whereas the maximum likelihood estimate, already discussed, can be represented in the basic framework by appropriate selection of coefficients, a maximum *a posteriori* (MAP) estimator requires the addition of an offset term to properly represent the prior. The equation for such an estimator was given by Kirlin as:

$$\begin{array}{c} {\hat{\Phi }}_{MAP}=\frac{\sum_{n=1}^{m}\left(2n-1\right)F_{n}/\sigma_{n}^{2}+\mu_{0}/\sigma_{0}^{2}}{\sum_{i=1}^{m}\left(2i-1\right)/\sigma_{i}^{2}+1/\sigma_{0}^{2}}. \end{array}$$ (21)

The variables *μ*_0_ and $\sigma_{0}^{2}$, in this case, represent the mean and variance of Φ, respectively. After manipulating the terms, it can be found that

$$\begin{array}{c} \beta_{n}=\frac{{\left(2n-1\right)}^{2}}{\sigma_{n}^{2}\sum_{i=1}^{m}{\left(2i-1\right)}^{2}/\sigma_{i}^{2}+1/\sigma_{0}^{2}}, \end{array}$$ (22)

and the offset term can be expressed as

$$\begin{array}{c} \beta_{0}=\frac{\mu_{0}/\sigma_{0}^{2}}{\sum_{i=1}^{m}{\left(2i-1\right)}^{2}+1/\sigma_{0}^{2}}. \end{array}$$ (23)

To assess the utility of this extended model from Eq (20), the experiments described in the section Length Estimation Experiments were repeated on both simulated and human speech data, with coefficients determined according to Eq (22) and offset determined according to Eq (23). The results of these experiments can be seen in Tables 2 and 3 (the estimator called *Max. a Post.*). This estimator was found to provide an RMS error of 0.671 cm on simulated speech data and 0.946 cm on human speech data. Both of these accuracies are superior to the performance of any of the previously described estimators on their respective data sets. It is expected, in general, that a more complex model will be able to provide better performance by allowing a better fit to the data.

Note also that Eq (20) constitutes a multiple linear regression model that, like the model presented in Eq (4), can be fit easily using a standard analytical solution. Therefore, it is also possible to stay within the spirit of the estimator proposed in this work, and to fit this slightly more complex linear model by finding the least squares solution. To that end, the experiments described in the section Length Estimation Experiments were repeated on both simulated and human speech data, with coefficients determined by finding the least-squares solution. The results of these experiments can be seen in Tables 2 and 3 (the estimator called *Proposed—Ext.*), and they show that this estimator was found to provide an RMS error of 0.608 cm on simulated speech data and 0.839 cm on human speech data. These estimation results were represent the best performance observed in the course of the present work on their respective data sets. Note, also, that the coefficient values in this estimator increase as formant number increases, which is again consistent with the relative insensitivity of higher formants.

## Conclusion

A general framework for designing vocal tract length estimators was developed, beginning from the basic principles of vocal tract acoustics. It was shown that several previously proposed estimators fit within this framework. Moreover, it was shown that an estimator which is optimal in the least-squares sense can be developed in a statistical fashion with a sufficient amount of data using multiple linear regression. The proposed estimator was evaluated on both simulated and human speech data sets and was shown to outperform previously proposed estimators. A key characteristic of the proposed estimator was the increasing weight placed on higher formants, suggesting that higher formants are more reliable features for estimating vocal tract length. Direct examination of formant frequency variation in the current data sets further reinforced the idea that higher formants are less sensitive to speech articulation. These empirical findings lead to a theoretical examination of formant sensitivity, where corroborating predictions from two methods of vocal tract acoustic modeling were found. Insights from perturbation theory further revealed that vocal tract constrictions can be interpreted as filtering the formant sensitivity functions, and the frequency response of this filter generally decreases with frequency.

Vocal tract length estimation is an instance of morphological inversion of speech—that is, attempting to predict inherent speaker-specific characteristics about the size and shape of the speech production apparatus from the acoustic signal. Morphological inversion holds promise for many technological applications where knowledge of morphological characteristics would be advantageous in analyzing the speech signal. In addition to normalizing acoustic characteristics of different speakers, accurate length estimation could lead to biometric applications, because vocal tract length may represent an individual speech characteristic that cannot be easily forged or altered. An interesting extension of the present work will be to further examine vocal tract length estimation from the dynamic perspective concerning vocal tract length. Data from rtMRI will allow us to measure vocal tract length at finer temporal scales in the future, and to do so with higher accuracy than previously possible. It would be interesting to see how the various models discussed here will perform when data concerning vocal tract length comprise precise, short-time length measurements. It will also be interesting to examine the perceptual aspects of vocal tract length estimation in humans to provide deeper insights into the success of length normalization in technological applications, and to examine situations where perception of vocal tract length can apparently be misleading or inaccurate. For instance, Black-and-White Colobus monkeys produce vocalizations that come from apparently much longer vocal tracts than they actually possess [26], and it has been suggested that human males have vocal tracts optimized to give the impression of size [48], but presumably without compromising intelligibility.
